# The impact of a digital guideline version on schizophrenia guideline knowledge: results from a multicenter cluster-randomized controlled trial

**DOI:** 10.1186/s12916-024-03533-6

**Published:** 2024-07-29

**Authors:** Theresa Halms, Gabriele Gaigl, Carolin Lorenz, Duygu Güler, Naiiri Khorikian-Ghazari, Astrid Röh, Angelika Burschinski, Wolfgang Gaebel, Marisa Flick, Charline Pielenz, Eva Salveridou-Hof, Thomas Schneider-Axmann, Marco Schneider, Elias Wagner, Peter Falkai, Susanne Lucae, Michael Rentrop, Peter Zwanzger, Florian Seemüller, Michael Landgrebe, Marion Ortner, Bertram Schneeweiß, Peter Brieger, Klemens Ajayi, Michael Schwarz, Stephan Heres, Nicolay Marstrander, Thomas Becker, Markus Jäger, Albert Putzhammer, Karel Frasch, Raimund Steber, Stefan Leucht, Alkomiet Hasan

**Affiliations:** 1 Department of Psychiatry, Psychotherapy and Psychosomatics, University of Augsburg, Medical Faculty, BKH Augsburg, Geschwister-Schönert-Str. 1, Augsburg, 86156 Germany; 2 Department of Psychiatry and Psychotherapy, Technische Universität München, Medical Faculty, Klinikum rechts der Isar, Munich, Germany; 3 Department of Psychiatry and Psychotherapy, LVR-Klinikum Düsseldorf, Medical Faculty, Heinrich-Heine-University, Düsseldorf, Germany; 4 WHO Collaborating Centre on Quality Assurance and Empowerment in Mental Health, Düsseldorf, EU131 Germany; 5 Department of Psychiatry and Psychotherapy, University Hospital, LMU Munich, Munich, Germany; 6 Evidence-Based Psychiatry and Psychotherapy, Faculty of Medicine, University of Augsburg, Augsburg, Germany; 7 Max Planck Institute of Psychiatry, Munich, Germany; 8 Kbo Inn-Salzach-Klinik Wasserburg, Wasserburg, Germany; 9 Kbo-Lech-Mangfall-Klinik Garmisch-Partenkirchen und Peißenberg, Garmisch-Partenkirchen, Germany; 10 Kbo-Lech-Mangfall-Klinik Agatharied, Hausham, Germany; 11 Kbo-Lech-Mangfall-Klinik Landsberg am Lech, Landsberg am Lech, Germany; 12 Kbo-Klinik Taufkirchen/Vils, Taufkirchen, Germany; 13 Kbo-Isar-Amper-Klinikum München-Ost, Munich, Germany; 14 Kbo-Isar-Amper-Klinikum München-Nord, Munich, Germany; 15 Department of Psychiatry and Psychotherapy II, Ulm University, BKH Günzburg, Günzburg, Germany; 16 Department of Psychiatry and Psychotherapy, University of Leipzig, Leipzig, Germany; 17 BKH Kempten, Kempten, Germany; 18 BKH Kaufbeuren, Kaufbeuren, Germany; 19 BKH Donauwörth, Donauwörth, Germany; 20 BKH Memmingen, Memmingen, Germany; 21 DZPG (German Center for Mental Health), partner site München/Augsburg, Munich/Augsburg, Germany

**Keywords:** Health personnel, Practice guideline, Cluster-randomized controlled trial, Guideline implementation, MAGICapp

## Abstract

**Background:**

Clinical practice guidelines are crucial for enhancing healthcare quality and patient outcomes. Yet, their implementation remains inconsistent across various professions and disciplines. Previous findings on the implementation of the German guideline for schizophrenia (2019) revealed low adherence rates among healthcare professionals. Barriers to guideline adherence are multifaceted, influenced by individual, contextual, and guideline-related factors. This study aims to investigate the effectiveness of a digital guideline version compared to print/PDF formats in enhancing guideline adherence.

**Methods:**

A multicenter, cluster-randomized controlled trial was conducted in South Bavaria, Germany, involving psychologists and physicians. Participants were divided into two groups: implementation of the guideline using a digital online version via the MAGICapp platform and the other using the traditional print/PDF version. The study included a baseline assessment and a post-intervention assessment following a 6-month intervention phase. The primary outcome was guideline knowledge, which was assessed using a guideline knowledge questionnaire.

**Results:**

The study included 217 participants at baseline and 120 at post-intervention. Both groups showed significant improvements in guideline knowledge; however, no notable difference was found between both study groups regarding guideline knowledge at either time points. At baseline, 43.6% in the control group (CG) and 52.5% of the interventional group (IG) met the criterion. There was no significant difference in the primary outcome between the two groups at either time point (T0: Chi^2^_(1)_ = 1.65, *p* = 0.199, T1: Chi^2^_(1)_ = 0.34, *p* = 0.561). At post-intervention, both groups improved, with 58.2% in the CG and 63.5% in the IG meeting this criterion.

**Conclusions:**

While the study did not include a control group without any implementation strategy, the overall improvement in guideline knowledge following an implementation strategy, independent of the format, was confirmed. The digital guideline version, while not superior in enhancing knowledge, showed potential benefits in shared decision-making skills. However, familiarity with traditional formats and various barriers to digital application may have influenced these results. The study highlights the importance of tailored implementation strategies, especially for younger healthcare providers.

**Trial registration:**

https://drks.de/search/de/trial/DRKS00028895

**Supplementary Information:**

The online version contains supplementary material available at 10.1186/s12916-024-03533-6.

## Background

Clinical practice guidelines are defined as “(…) systematically developed statements to assist practitioner and patient decisions about appropriate health care for specific clinical circumstances.” [1]. When implemented effectively, clinical practice guidelines were shown to improve the process of care as well as patient outcomes [2]. However, guideline implementation still remains insufficient—across countries, disciplines, and professions [3–7]. Specifically, a recent study on the implementation of the German evidence- and consensus-based guideline for schizophrenia (2019) [8] demonstrated that less than 50% of the surveyed healthcare professionals were aware of and accepted the content of the guideline as a whole, and less than 10% reported routinely applying the recommendations to eligible patients [6]. Regarding specific aspects of the guideline, awareness (38%), agreement (36%), adoption (33%), and adherence (5%) were lowest for one recommendation regarding the management of severe weight gain, whereas one recommendation concerning antipsychotic relapse prevention received the highest rates of awareness (81%), agreement (88%), adoption (74%), and adherence (40%) [6].

One systematic review with a meta-analysis of 19 studies on the impact of guideline implementation on practitioner performance and different patient outcomes revealed that only few studies showed an effect on provider performance [9]. Moreover, small to medium effects of guideline implementation on different patient outcomes, such as clinical condition, remission rate, and satisfaction with care, were found [9]. Thus, the lack of scientific evidence regarding the effectiveness of guideline implementation as well as possible strategies to increase the effectiveness and adherence of guidelines become apparent.

In addition, a qualitative study including a total of 30 practitioners from the Netherlands showed that 46% of the participants considered a lack of knowledge regarding guideline recommendations to be a key barrier to adherence [10]. These findings were confirmed by a systematic meta-review of twelve systematic reviews, which demonstrated that familiarity with the content of guideline recommendations contributes considerably to their successful implementation [11]. Another possible reason for barriers in guideline implementation may be the exponential growth in medical knowledge and subsequently in recommended treatment options. One study conducted among 400 physicians revealed that the most frequently mentioned barriers to guideline adherence were the complexity of guidelines, a high number of weak recommendations, and lack of time due to clinical obligations [12]. As a result, guidelines are not only becoming increasingly multilayered, but are often partially out of date by the time they are published. Therefore, it is a crucial next step to adapt the publication form of guidelines to the constant and rapid progress in clinical research.

In that regard, living guidelines are defined as an optimized guideline development process by allowing updates of recommendations as soon as new and relevant evidence becomes available [13] or at least annually [14]. To facilitate seamless updates, advanced systems for guideline development have been created, embracing a digital, web-based, and platform-independent approach. The MAGICapp is one possible and frequently used web-based tool for authoring, publishing, and updating digitally structured clinical practice guidelines [15]. MAGICapp, the currently most widely used platform [16], is based on the GRADE approach (Grading of Recommendations Assessment, Development and Evaluation) [17] and allows a visual presentation of the evidence and offers novel possibilities for shared decision-making. In addition, transparency and continuity are ensured by linking corresponding and recently published articles to the recommendations as well as related recommendations from other guidelines to gain deep insight into the data and facilitate well-informed decision-making [18].

Despite the growing interest in the concept of digital guidelines and the expected increase in utilization of the guideline if continuously updated [13], little to no evidence regarding the impact of a digital guideline implementation on guideline knowledge is available. Our aim was to evaluate whether a newly developed digital guideline concept of the German S3 guideline for schizophrenia (2019) (DGPPN 2019), implemented in the evidence-ecosystem MAGICapp, is superior to the print form in terms of guideline knowledge of mental health care professionals. Therefore, we conducted a multicenter, controlled, cluster-randomized two-arm trial investigating the implementation of the German schizophrenia guideline among medical doctors and psychologists in a 6-month study period. We hypothesized that the gain of guideline knowledge will be higher in those healthcare professionals who had access to the digital evidence-ecosystem.

## Methods

### Study design and randomization

This study is a multicenter, cluster-randomized controlled trial addressing psychologists and physicians employed at 17 hospitals or departments for psychiatry and psychotherapy in a model region in South Bavaria, Germany. The methods and study procedures have been described in detail in the published study protocol [19]. In the interventional group (IG), the implementation of the German schizophrenia guideline was conducted using a digital online version via the MAGICapp platform, while in facilities belonging to the control group (CG), implementation was carried out using the classic print or PDF version of the guideline. Cluster randomization was established to avoid interactions between subjects from different groups within one hospital. The participating 17 hospitals were organized into *n* = 14 clusters based on their respective number of employees. Randomization was performed by a blinded statistician (TSA) experienced in the method of the Mersenne-Twister algorithm [19]. The baseline survey took place from 05/2022 to 06/2022, followed by a 6-month implementation phase of the German schizophrenia guideline from 06/2022 to 11/2022. Subsequently, the post-intervention survey was conducted from 11/2022 to 01/2023. The study was reviewed by the data protection officer of the University Hospital Munich (LMU Munich) and approved by the ethics committee of the Medical Faculty of the Ludwig-Maximilians-University (LMU) Munich, Germany (Ref. 21-0780) and was performed in accordance with the ethical standards as defined in the Declaration of Helsinki. The participants provided their written informed consent to participate in this trial.

### Intervention

During the 6-month study phase, the German schizophrenia guideline was implemented in the interventional group (IG) using a digital online version within the evidence-ecosystem MAGICapp (for example, see Additional file 1: Figs. 1–6), prior to study start the complete guideline was transferred to the MAGICapp and designed accordingly, while the control group (CG) only had access to the current PDF or print version. Participants of the IG had access to the guideline implemented in MAGICapp using login credentials provided by the study team. In contrast to the PDF version, the guideline embedded in MAGICapp provided advanced navigation features, such as hyperlinks, filters, and interactive elements as well as visual presentations of evidence. Further, the MAGICapp featured links to related recommendations from other guidelines and relevant publications and included interactive charts facilitating shared decision-making as well as decision aids [15]. The PDF provided to the CG was a static document 100% comparable to the content of the guideline published as book version. At the beginning of the implementation phase, participants of the intervention group received training on the principles and aims of a digital guideline platform such as MAGICapp as well as an interactive hands-on training on the use and functions of the digital online version of the German schizophrenia guideline in MAGICapp. In the CG, participants only received the freely accessible long version of the guideline in PDF format [14] as well as a training in its application. For both groups, digital expert boards were held by Stefan Leucht and Alkomiet Hasan every other week via video conferences, in which questions and suggestions of the study participants as well as different key topics of the guideline were discussed.

### Outcomes

As a primary outcome, guideline knowledge was assessed using a total of 46 knowledge questions including five cardinal questions of particular importance. This questionnaire, which was developed by the study team (AH, GG, TH, AR, NG), was based on previously published results of a study on physicians’ compliance with guidelines for the treatment of cardiovascular diseases [20]. All questions were designed as five-choices multiple-choice questions referring to case vignettes developed by the study team. In accordance with the questionnaire used by Karbach et al. [20], the questions investigated both a quantitative aspect (number of answers appropriate regarding the contents of the guideline) as well as a qualitative aspect (five cardinal questions). To prevent learning effects, the order of case vignettes as well as names and gender of the fictional patients mentioned in the case vignettes were modified from baseline to post-survey. Guideline knowledge was operationalized as providing at least 30 out of 46 correct answers, including all five cardinal questions. The latter were defined by AH and SL and correspond to guideline recommendations with the highest level of evidence with a paramount importance for schizophrenia care. An error in one or more of the cardinal questions was per se considered as non-adherent regardless of performance in other questions. Drop-outs were defined as participants who did not answer any questions of the guideline knowledge questionnaire in the post-survey.

Secondary outcomes included the digital health expertise measured with the eHealth literacy scale [21], usability of the respective formats by the “System Usability Scale” [22], caregivers’ confidence in decision-making measured via the “Provider Decision Process Assessment Instrument” as well as the ability in shared decision-making using the “Participatory Decision Making Questionnaire” (PEF-FB-Doc) [23, 24]. Furthermore, attitudes towards and use of the respective guideline version were measured using a questionnaire developed by the authors for this trial (see Additional file 2: Table 1). For all scales, if necessary, variables were coded in a way that higher values were always better than lower values.

### Power analysis

Anticipating an increase in participants meeting the primary outcome from 40% in the classical print version group to 60% in the MAGICapp group, we determined that a total sample size of *N* = 237 was necessary. This calculation considered a significance level of *α* = 0.05, a power of 1 − *ß* = 0.8, a two-sided Fisher’s exact test as the analysis method, and a cluster correction factor of 1.16. Please see the published protocol for all details [19]. The achieved power ultimately exceeded 1 − *ß* = 0.8, allowing for a detectable difference between the groups of Δ0 = 20% at baseline and Δ1 = 26% at T1. These calculations assumed likelihood ratio chi-square tests for the performed analyses.

### Statistical analysis

A statistical analysis plan was developed prior to all analyses by TSA. All statistical analyses were conducted using a blinded approach through blinding of the statistician until analyses were performed. All analyses were carried out using IBM SPSS for Windows (version 28) with a significance level of *α* = 0.05. Primary analyses were performed for the intention-to-treat sample (ITT sample), including all participants as randomized that answered at least one knowledge question at baseline. For baseline (T0) and post-intervention (T1) demographic data, descriptive statistics including the frequency of drop-outs were assessed separately for the intervention and the interventional group and compared between both groups using Mann-Whitney *U* tests and likelihood ratio Chi^2^ (LR Chi^2^) tests. In case of cells with less than five participants, Fisher-Freeman-Halton exact test was computed instead.

The primary analysis was a comparison of guideline compliance in terms of knowledge on the German schizophrenia guideline (≥ 30 correctly answered questions and five correctly answered cardinal questions) between the intervention and the control group using LR Chi^2^ tests among the intention-to-treat sample. Therefore, LR Chi^2^ tests were applied to test the hypothesis for the primary outcome. In case of significant differences, Cramér’s *V* was calculated to determine effect sizes. To test for homogeneity of the odds ratios, Breslow-Day test was used and Cochran test was applied to analyze conditional independence. For the secondary outcome of correctly answered questions, the same set of 46 knowledge questions was assessed for each participant and compared between the IG and CG using LR Chi^2^ tests. However, this secondary analysis considered a broader view of changes in guideline knowledge without the requirement of correctly answering all five cardinal questions. For continuous secondary outcome variables, deviations from the normality assumption were identified through the Kolmogorov-Smirnov test. Despite employing appropriate variable transformations, these deviations persisted. Consequently, non-parametric tests, specifically Mann-Whitney *U* tests for group comparisons and Wilcoxon tests for paired data at T0 and T1, were employed.

## Results

### Demographics

**Fig. 1:**
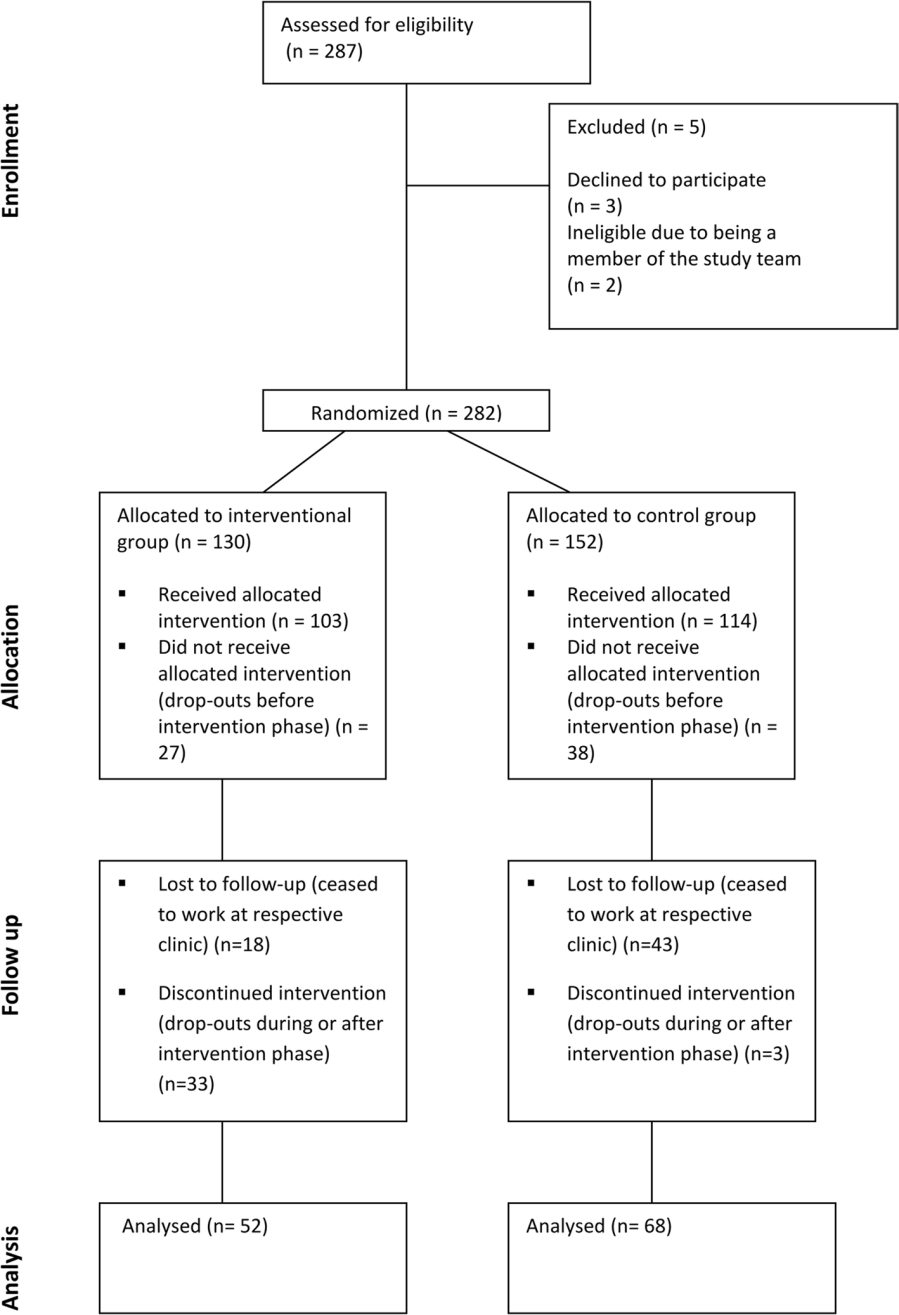
CONSORT flow chart showing enrollment and participation in cluster-randomized trial

Initially, 287 healthcare professionals who responded to the invitations to participate in the study were assessed for eligibility. Out of these, three subsequently declined to participate and two were unsuitable due to being a member of the study team. Further, a total of 65 practitioners did not complete the baseline questionnaire and therefore dropped out before the intervention phase (CG: *n* = 38, IG: *n* = 27). At T0, a total of *N* = 217 mental health care professionals completed the questionnaire (baseline assessment) (CG: *n* = 114, IG: *n* = 103). At T1 (post-intervention assessment), *N* = 120 respondents completed the survey (CG: *n* = 68, IG: *n* = 52), resulting in a total drop-out number of *n* = 97 participants. Out of these 97 drop-outs, *n* = 61 participants ceased to work at their initial employer clinic during the study period and therefore did not take part in the post-survey. See Fig. 1 for a flow diagram of participants. Statistics revealed no significant differences between the control and interventional groups for age (*F*_(1, 215)_ = 2.64, *p* = 0.106) or for gender distribution (Chi^2^_(1)_ = 2.33, *p* = 0.127). Further, there were no significant differences between both groups regarding years of work experience (*F*_(1, 202)_ = 2.05, *p* = 0.154) and profession (Chi^2^_(9)_ = 10.16, *p* = 0.337) and type of hospital (university hospital/non-university hospital) where the study participants were employed (Chi^2^_(1)_ = 0.31, *p* = 0.575). See Table 1 for descriptive data and test statistics.

**Table 1:** Demographic and occupational characteristics of study sample at baseline

	**Total**			**CG**			**IG**			***F***	**df**	***p***
	***N***	**M**	**SD**	***N***	**M**	**SD**	***N***	**M**	**SD**			
**Age (in years)**	217	37.18	9.32	114	36.20	8.76	103	38.25	9.84	2.64	1	0.106
**Work experience (in years)**	204	8.24	7.58	108	7.52	6.80	96	9.04	8.34	2.05	1	0.154
	***N***	**%**		***N***	**%**		***N***	**%**		**X**^**2**^	***p***	
**Gender**										2.33	0.127	
Male	63	29.03		28	24.56		35	33.98				
Female	154	70.97		86	75.44		68	66.02				
**Profession**										10.16	0.337	
*Psychologist/psychotherapist*												
Total	81	37.33		40	35.09		41	39.81				
Psychotherapist	25	11.52		11	9.65		14	13.59				
Psychotherapist in training	36	16.59		20	17.54		16	15.53				
Psychologist	20	9.22		9	7.89		11	10.68				
*Physicians*												
Total	136	62.67		74	64.91		62	60.19				
Specialist in psychiatry and psychotherapy	56	25.81		25	21.93		31	30.10				
Assistant physician in psychiatry and psychotherapy	70	32.26		45	39.47		25	24.27				
Assistant physician in psychosomatic medicine and psychotherapy	3	1.38		1	0.88		2	1.94				
Specialist in neurology	4	1.84		2	1.75		2	1.94				
Assistant physician in neurology	1	0.46		1	0.88		0	0.00				
Assistant physician for general medicine	1	0.46		0	0.00		1	0.97				
Specialist physician in another field	1	0.45		0	0.00		1	0.97				
**Participation in expert boards**										0.17	0.682	
Participated at least once	69	57.5		38	55.88		31	59.62				
Never participated	51	42.5		30	44.12		21	40.38				
**Participation in training session**										0.14	0.705	
Participated/viewed the recording at least once	95	79.17		53	77.94		42	80.77				
Never participated/viewed the recording	25	20.83		15	22.06		10	19.23				

*CG* Control group, *IG* Intervention group, *N* Number (group size), *M* Mean, *SD* Standard deviation, *F F*-statistic, *df* Degrees of freedom, *Χ*^*2*^ Chi^2^-statistic, *p P* value

### Comparison of efficacy outcomes

#### Primary outcome

**Fig. 2:**
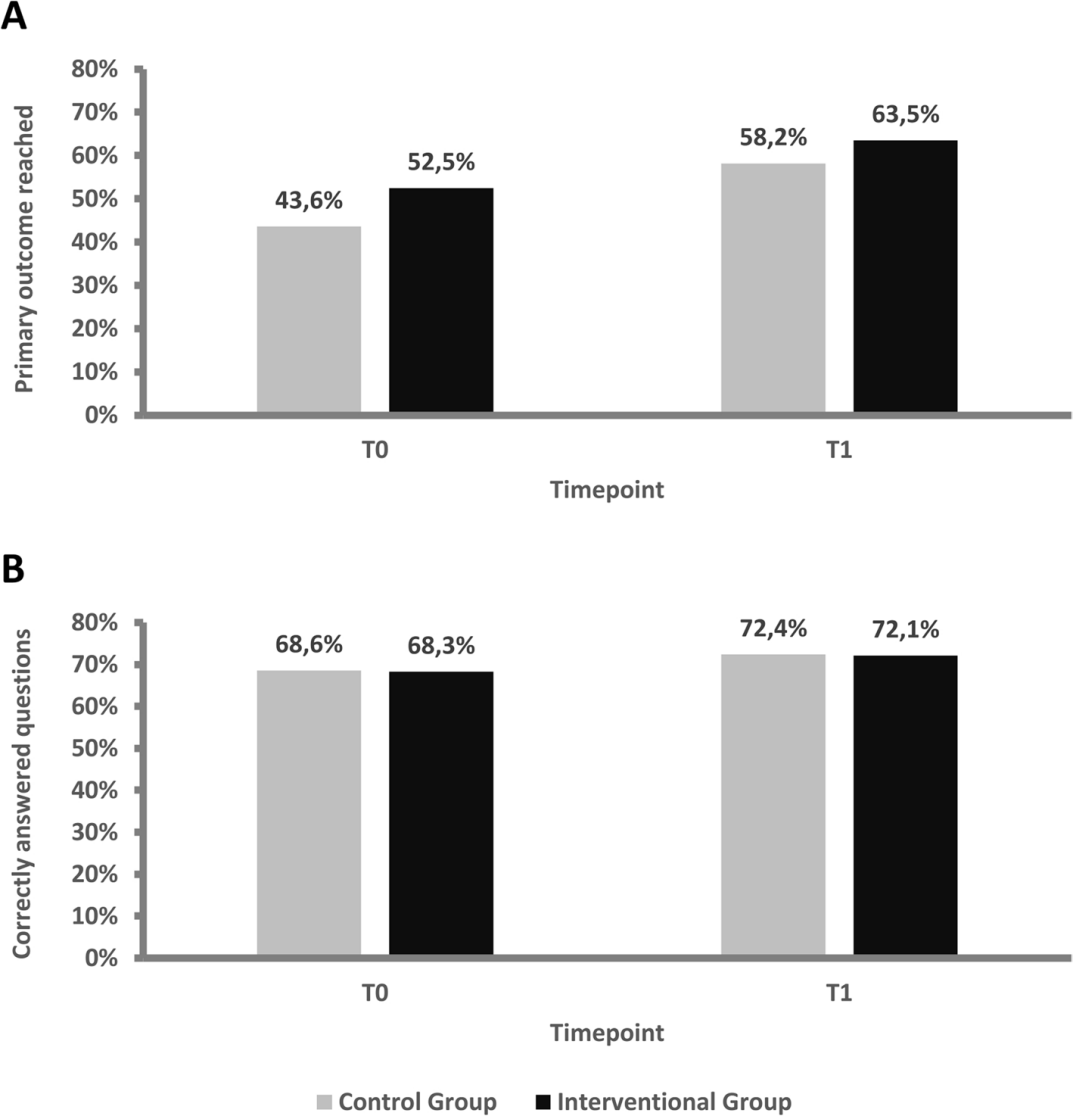
**A** Percentages of participants who reached the primary outcome at T0 and T1; **B** percentages of correctly answered questions among participants at T0 and T1

The primary outcome, guideline knowledge (criterion: at least 65% of questions correctly answered, all cardinal questions correctly answered), was compared between groups and time points using Chi^2^ tests and Breslow-Day tests. At baseline (T0), 43.6% of participants in CG and 52.5% of participants of IG met the criterion. At post-intervention (T1), both groups improved, with 58.2% in the control group and 63.5% in the IG meeting this criterion (see Fig. 2A). There was no significant difference in the primary outcome between the two groups at either time point (T0: Chi^2^_(1)_ = 1.65, *p* = 0.199, T1: Chi^2^_(1)_ = 0.34, *p* = 0.561). However, a notable imbalance between time points was detected (Chi^2^_(1)_ = 4.87, *p* = 0.027, *V* = 0.12), although not specifically for groups when examined individually (CG: Chi^2^_(1)_ = 3.54, *p* = 0.060; IG: Chi^2^_(1)_ = 1.68, *p* = 0.194, respectively). Moreover, improvement in the Breslow-Day test was not significantly different between groups (Chi^2^_(1)_ = 0.08, *p* = 0.775). See Additional file 2: Tables 2 and 3 for descriptive data and test statistics.

#### Secondary outcomes—correctly answered questions

Results of the analysis based on all 46 answered questions indicated that at time point T0, 44% of participants in the CG and 53% in the IG met the criterion of guideline knowledge. At time point T1, 58.2% in the CG and 63.5% in the IG met the criterion. Thus, an improvement was evident in both groups (CG: + 35.1%, IG: + 19.8%). Consequently, there was no significant imbalance in the criterion distribution between study arms at both time points (T0: Chi^2^_(1)_ = 1.52, *p* = 0.217, T1: Chi^2^_(1)_ = 0.15, *p* = 0.702). The improvement did not differ significantly between groups in the Breslow-Day test (Chi^2^_(1)_ = 0.17, *p* = 0.678). At T0 and T1, Mann-Whitney *U* tests revealed no differences in the percentages of correctly answered questions across groups (T0: *U* = 5843, *p* = 0.952, T1: *U* = 1737.5, *p* = 0.871). At T0, participants in the CG answered 68.6% and participants in the IG answered 68.3% of all questions correctly. At time point T1, participants in the CG reached 72.4% and participants in the IG reached 72.1% correctly answered questions (see Fig. 2B). At T0, there were significant differences in the attainment of the primary outcome between the professional groups, with the highest attainment (80%) in the group of specialist physicians in psychiatry and psychotherapy (Freeman-Halton = 49.89, *p* < 0.001). Further, years of professional experience were significantly higher in the group meeting the primary outcome than in the group not meeting the primary outcome at both time points (*U* ≥ 916, T0, T1: *p* < 0.001). Moreover, participation in digital expert boards was more frequent in the primary outcome attainment group than in the nonattainment group (Chi^2^_(1)_ = 8.87, *p* = 0.003, *V* = 0.27) meaning that greater participation in expert panels was associated with a higher likelihood of meeting the primary outcome. See Additional file 2: Table 4 for descriptive data and complete test statistics.

### Further secondary outcomes

Mann–Whitney *U* tests were used to compare variables (attitudes towards guideline formats, eHealth literacy, shared decision-making, and decision-making confidence) between groups at T0 and T1. Initially, the control group (CG) had higher digital health literacy than the intervention group (IG) (*U* = 4748, *p* = 0.015), but no significant differences were found at T1. At T0, CG participants showed more confidence in using the German schizophrenia guideline than IG (*U* = 4896, *p* = 0.026), and by T1, CG participants referred to the guideline more frequently in the past 6 months (*U* = 1377, *p* = 0.027). CG participants also felt more certain about treatment decisions at T1 (*U* = 1402, *p* = 0.036). Both groups improved in decision-making confidence and shared decision-making by T1, with significant increases in guideline use, confidence, and decision process aspects. However, the perceived regular usability of the print format declined in both groups (CG: *Z* =  − 3.53, IG: *Z* =  − 2.45, *p* < 0.001 and *p* = 0.014, respectively), and more participants needed assistance over time. IG showed significant improvements in guideline confidence, ease of use, and decision-making between T0 and T1, but found the print format less useful at T1 (*Z* =  − 2.96, *p* = 0.003). Attitudes towards the guideline formats were mixed; the print/PDF version was favored for confidence and support needs, while the MAGICapp format excelled in ease of use, function integration, and learning speed. Additional file 2: Tables 2, 3, and 5 provide detailed data and statistics.

For further secondary outcomes, see Additional file 2: Tables 2, 3, and 5.

## Discussion

The results of this multicenter, cluster-randomized, controlled trial demonstrated a significant improvement in guideline knowledge in both study groups during a 6-month implementation phase. The positive impact of the implementation strategy, which consisted of training courses on the use of the guideline as well as participation in expert boards, was demonstrated. The effectiveness of multifaceted interventions and especially educational measures was previously confirmed by an umbrella review based on a total sample size of 22,512 health care professionals, which showed that interactive learning units, workshops, and hands-on training sessions at regular intervals were particularly effective regarding the implementation of clinical practice guidelines [25]. Additionally, one scoping review including 118 studies on the implementation of guidelines revealed that nearly 56% of studies reported implementation strategies to be effective on practitioners’ actions in clinical practice, knowledge, attitudes, or multiple of these outcomes, highlighting the positive impact of interventions on guideline knowledge and adherence [26]. Additionally, one implementation study conducted in Sweden in six psychiatric hospitals demonstrated a significant improvement of compliance to clinical guidelines for the treatment of depression and suicidal behaviors following an active implementation phase of 24 months in total [27]. The study further showed that while improvement in the intervention group receiving the implementation phase was significant, guideline adherence neither improved nor declined among the participants of the control group [27]. While our here presented trial did not include a control group without any interventions, the observed improvements in guideline knowledge among both the intervention and control group highlight the positive impact of structured guideline implementation. The positive effects of guideline implementation strategies have previously been confirmed by several studies demonstrating the effectiveness of structured implementation strategies such as interactive training and expert consultations.

However, we were not able to establish significant differences between the group receiving the PDF/print and the group using the digital guideline version in MAGICapp. One possible reason may be the novelty of the presentation and functions in MAGICapp, which may initially require a longer adjustment phase. Moreover, as the MAGICapp was not embedded in the hospital information systems, the need to switch to a web browser may have also been a limitation. Further, the use of digital applications may present novel barriers among a subgroup of healthcare professionals, which may in turn impact the acquisition of guideline knowledge. One systematic review comprising 108 studies on barriers and facilitators to the use of digital health technologies among healthcare professionals showed that the main hindering factors were technical adversities and barriers regarding infrastructure [28]. These include, for instance, lack of network, insufficient technologies and devices, slow or unstable internet connectivity, and poor compatibility with existing work processes [28]. Other major obstacles identified were personal and psychological barriers, including fear of technology, difficulties in understanding and using new digital applications, and a general resistance to change [28]. We cannot disentangle which of these factors contribute to our finding, but in summary, the digital version was not superior in terms of implementation success.

Our analyses revealed that other factors, such as belonging to the professional group of specialist physicians in psychiatry and psychotherapy as well as years of professional experience, were associated with higher guideline knowledge. These results reflect the findings of our previously conducted survey on the implementation status of the German schizophrenia guideline, which revealed that specialist physicians demonstrated higher awareness, agreement, and adoption of the guideline compared to other mental health care professionals [3, 6]. Moreover, the higher guideline adherence among specialist physicians shown in our study may be caused by the overall number and specificity of recommendations for physicians compared to other professions in the guideline. Our results revealed that both older participants as well as participants with more years of professional experience had a higher level of guideline knowledge than health care professionals of a lower age and with less working experience. Cumulative clinical experience over the course of time may contribute to a deeper understanding of the schizophrenia guideline on the one hand, as well as the ability to apply guideline recommendations to diverse patient cases on the other hand. Concurrently, one systematic meta-review synthesizing the results of twelve systematic reviews found that younger practitioners and health care professionals with less professional experience were more inclined to use clinical practice guidelines than older medical staff with more years of experience [11], suggesting that implementation strategies specifically adapted to younger health care providers may be particularly relevant. Given that our previous study showed attitudes towards a living guideline to be mostly positive, especially among younger professionals [6], a digital app-based version of the German S3 guideline schizophrenia may serve as a key implementation tool, especially for early career practitioners.

Moreover, digital guideline versions facilitate patient involvement, for instance by providing presentation options that promote shared decision-making, such as those embedded in MAGICapp. Our study confirmed this showing significant improvements regarding shared decision-making skills among the participants of the IG.

### Limitations

Several limitations should be acknowledged. The drop-out rate, largely attributed to participants leaving their initial employer, may introduce selection bias. Additionally, disadvantages associated with online surveys, such as survey fraud and response bias, need to be considered. However, due to the restrictions imposed by the COVID-19 pandemic in the participating hospitals, conducting the present study on-site as initially planned was not possible. Further, the 6-month study duration may not capture long-term effects, especially when considering the required time to become familiar with the novel digital version of the guideline. It cannot be ruled out improvement over time may also be explained by learning effects. Nevertheless, we addressed this limitation by modifying the cases and re-arranging the order of questions between the two assessment points. Furthermore, participants were not provided with sample solutions, which may reduce learning effects. Another limitation that must be noted concerns the fact that guideline knowledge, which was assessed as the primary outcome of this study, may not necessarily reflect guideline adherence in clinical practice. Nevertheless, in the context of self-reported questionnaires, the assessment of knowledge on guideline recommendations represents an objective measure compared to self-reported adherence, thus minimizing the risk of bias as a consequence of socially desirable information. Further, it may be postulated that an increase in knowledge can be regarded as a consequence of guideline application and therefore function as a reflection of guideline adherence. However, this assumption requires further research to investigate the impact of a digital version of the schizophrenia guideline using MAGICapp on guideline adherence in clinical practice.

Moreover, the accessibility of the digital guideline format via MAGICapp was exclusively possible via browser and not embedded in the hospital information system, which made the setting less user-friendly. This limitation emphasizes the importance of contextual factors and infrastructure considerations in the implementation of online interventions. Additionally, due to data protection regulations and provisions of local staff councils, we were not allowed to verify that participants of the IG logged in and actively used the MAGICapp platform. However, MAGICapp use was actively promoted through the interactive hands-on training at the start of the intervention as well as the bi-weekly expert boards. Furthermore, the digital nature of the guideline that will form the basis for a living guideline raises concerns about the generalizability of our findings to healthcare professionals who may have varying degrees of familiarity with digital tools or preferences for traditional, offline resources. In addition, the online format may unintentionally exclude a subgroup of health care professionals less comfortable or experienced with digital tools, which could potentially lead to selection bias. Another limitation is the risk of imbalances between clusters due to the choice of a cluster-randomized design. Finally, our study was designed to assess guideline adherence in professionals, but we did not evaluate any patient-related (e.g., polypharmacy, remission rates) or socioeconomic-related (e.g., length of hospitals stays, overall treatment cross) endpoints.

### Conclusions

Overall, we were able to demonstrate a significant enhancement in guideline knowledge across both study groups following the 6-month implementation phase confirming that guideline implementation increases guideline knowledge. The efficacy of the implemented educational strategies, comprising training courses and participation in expert boards, highlights the positive impact of interactive training and regular practice related learning units, as supported by existing literature. However, the transformation into a digital guideline version was not superior with regard to the gain of knowledge. Furthermore, the association between increased guideline knowledge and age, professional experience, and the potential role of cumulative clinical experience emphasizes the need for tailored implementation strategies, particularly for younger health care providers. Future research should address longer follow-up periods to identify long-term effects and consider the preferences and needs of health care professionals, especially regarding the design and features of digital tools such as evidence-ecosystems. Finally, future clinical studies on guideline adherence in clinical practice in combination with the MAGICapp or other tools are needed.

### Supplementary Information

Additional file 1: Fig. 1. Table of contents of the German schizophrenia guideline in MAGICapp. Fig. 2. Example of evidence profiles for different outcomes in MAGICapp. Fig. 3. Graphic representation of PICO evidence profiles in MAGICapp. Fig. 4. Example of practical information embedded in MAGICapp. Fig. 5. Decision aids for shared decision-making in MAGICapp. Fig. 6. Features of MAGICapp.

Additional file 2: Table 1. Questionnaire assessing attitudes towards and use of the German schizophrenia guideline. Table 2. Primary and secondary outcomes CG vs. IG (at T0 and T1). Table 3. Primary and secondary outcomes T0 vs. T1 (for CG and IG). Table 4. Demographic/occupational characteristics for primary outcome (success vs. failed) among entire sample. Table 5. Secondary outcomes print format vs. MAGICapp (exclusively in the IG) at T1.

## Data Availability

The datasets used and/or analyzed during the current study are available from the corresponding author on reasonable request.

## Abbreviations

MAGIC: Making Grade the Irresistible Choice
GRADE: Grading of Recommendations Assessment, Development and Evaluation
CG: Control group
IG: Interventional group
LMU: Ludwig-Maximilians-University Munich
T0: Baseline assessment
T1: Post-intervention assessment
ADHD: Attention deficit hyperactivity disorder
