# Supplementary material for: The impact of a digital guideline version on schizophrenia guideline knowledge: results from a multicenter cluster-randomized controlled trial
Source: BMC Med. 2024 Jul 29;22:311. doi: 10.1186/s12916-024-03533-6 (PMC11287881; doi:10.1186/s12916-024-03533-6)
Supplement: Supplementary file 1 — Additional file 1: Fig. 1. Table of contents of the German schizophrenia guideline in MAGICapp. Fig. 2. Example of evidence profiles for different outcomes in MAGICapp. Fig. 3. Graphic representation of PICO evidence profiles in MAGICapp. Fig. 4. Example of practical information embedded in MAGICapp. Fig. 5. Decision aids for shared decision-making in MAGICapp. Fig. 6. Features of MAGICapp. [file 12916_2024_3533_MOESM1_ESM.docx]

**Figure 1: Table of contents of the German schizophrenia guideline in MAGICapp**


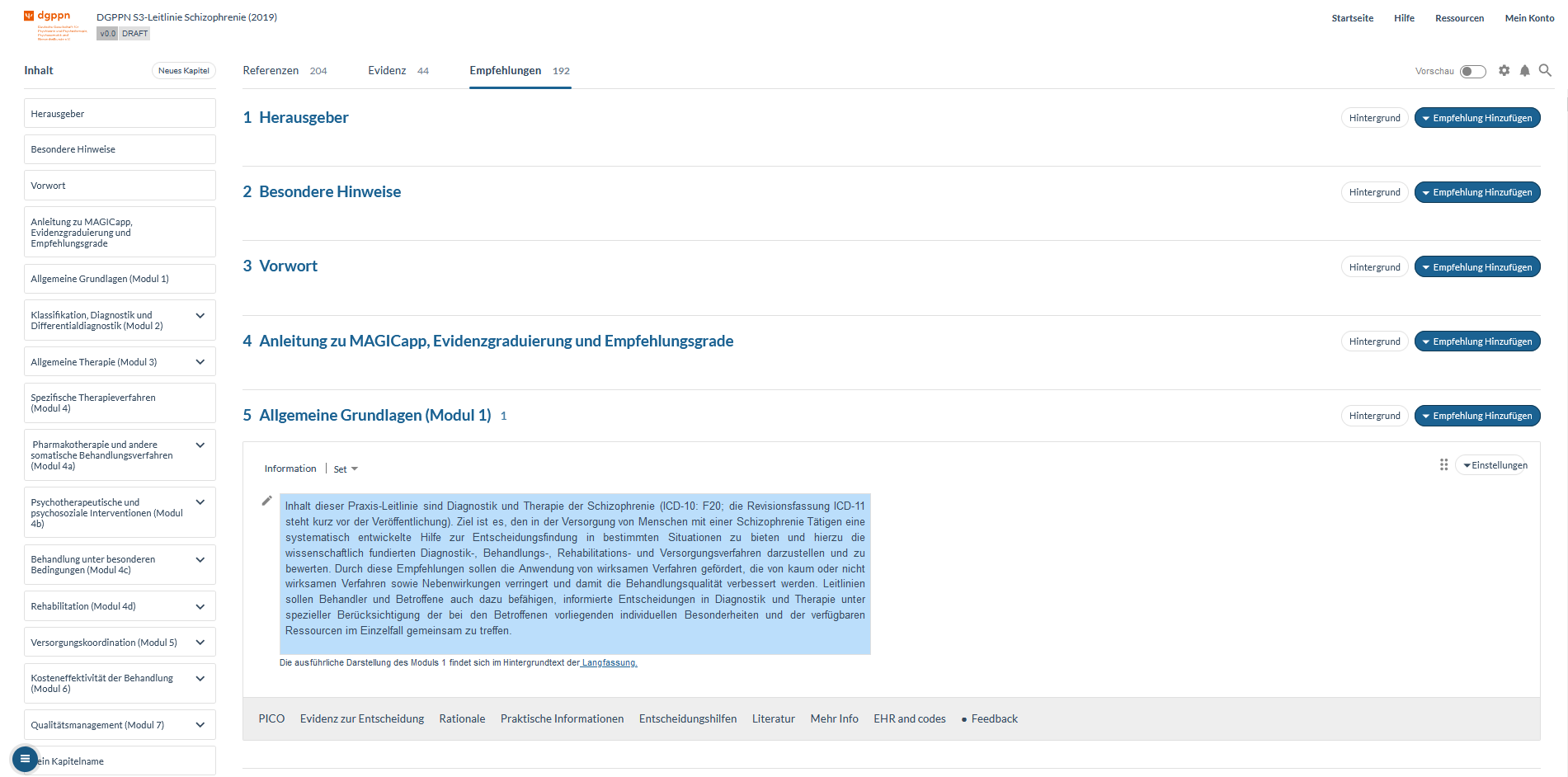


*From “www.app.magicapp.org S3-Leitlinie Schizophrenie* (2023) [cited 2023 May 18]. Not publicly available”.

**Figure 2: Example of evidence profiles for different outcomes in MAGICapp**


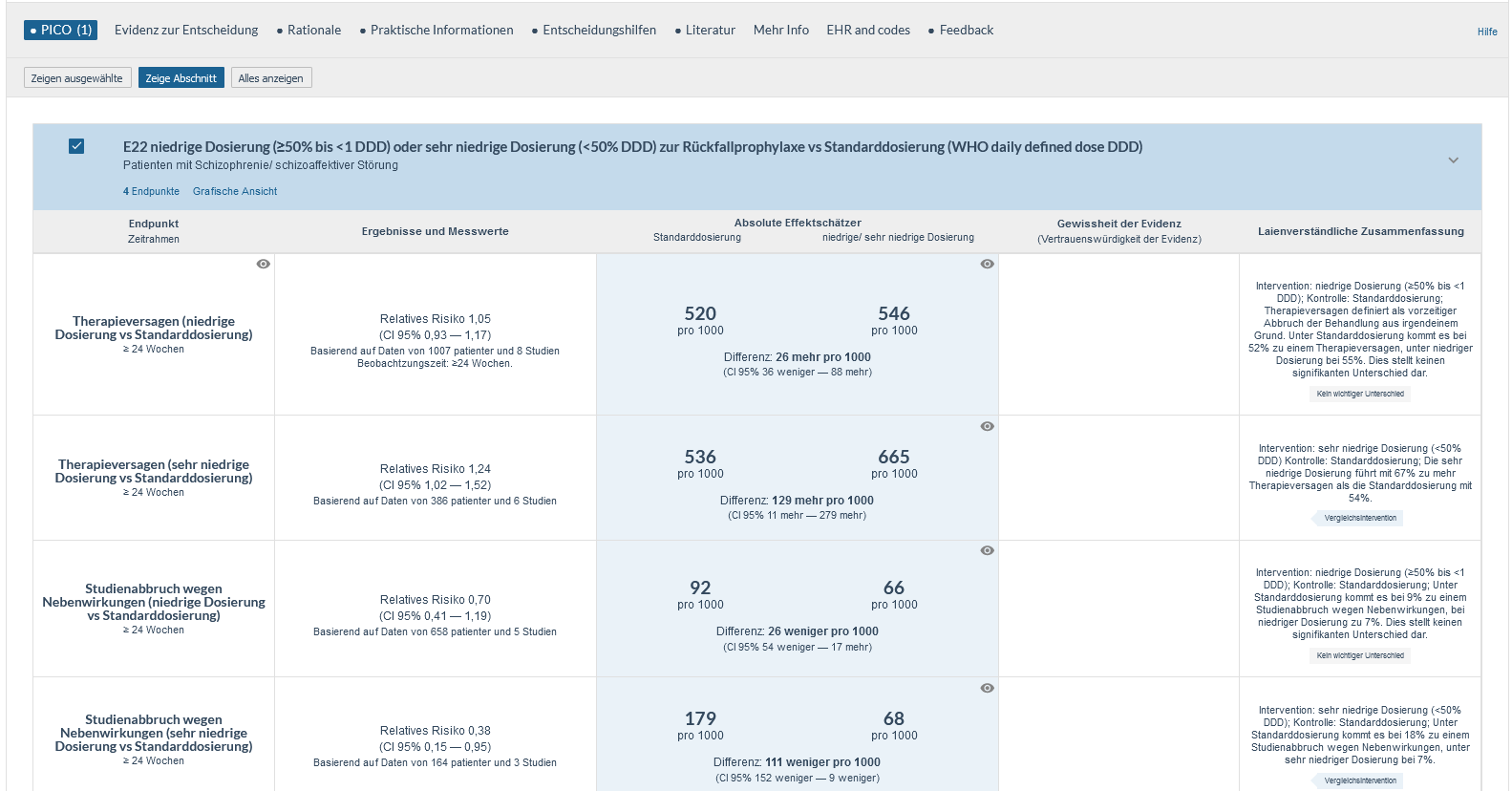


*From “www.app.magicapp.org S3-Leitlinie Schizophrenie* (2023) [cited 2023 May 18]. Not publicly available”.

**Figure 3: Graphic representation of PICO evidence profiles in MAGICapp**


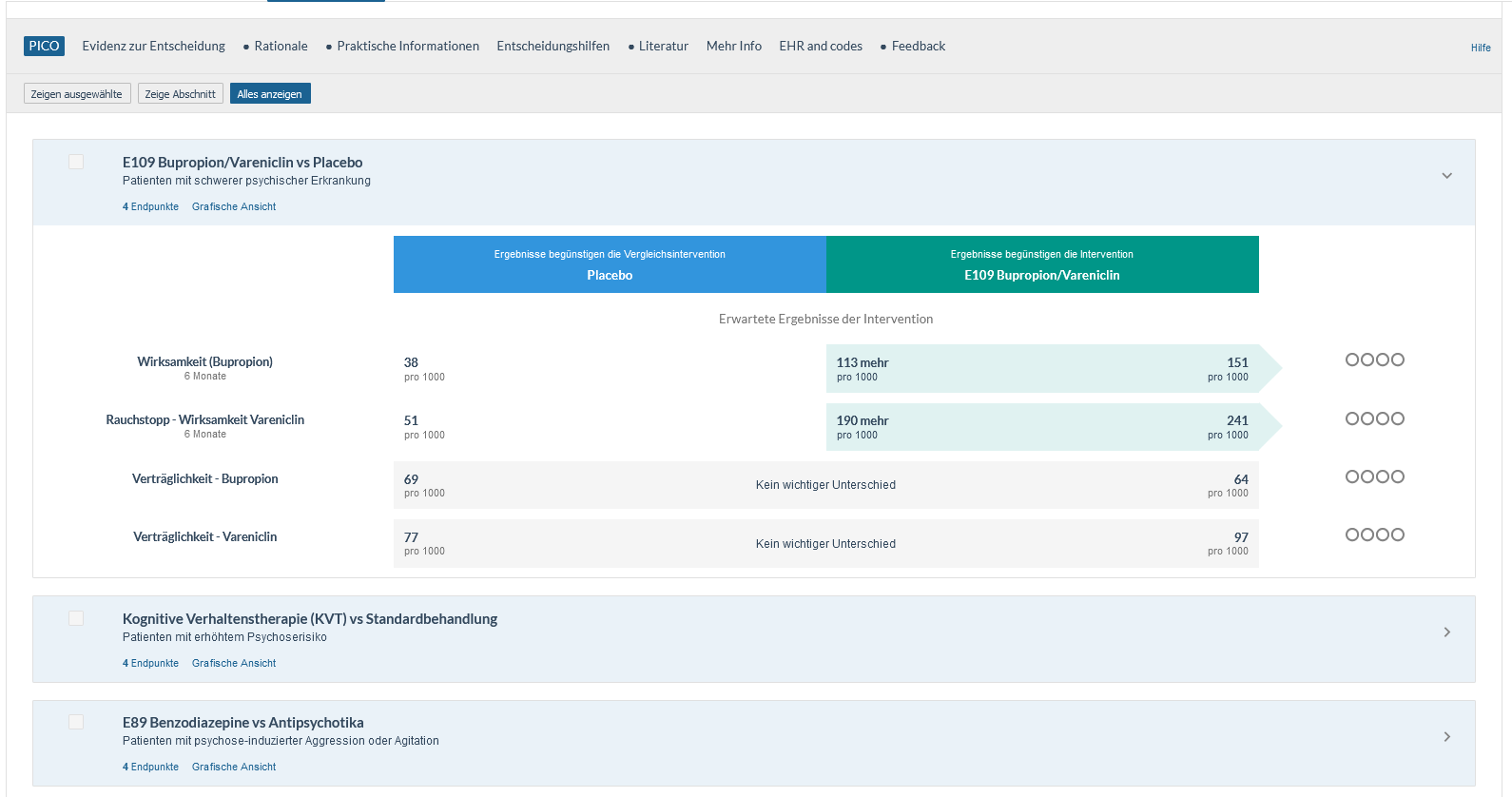


*From “www.app.magicapp.org S3-Leitlinie Schizophrenie* (2023) [cited 2023 May 18]. Not publicly available”.

**Figure 4: Example of practical information embedded in MAGICapp**


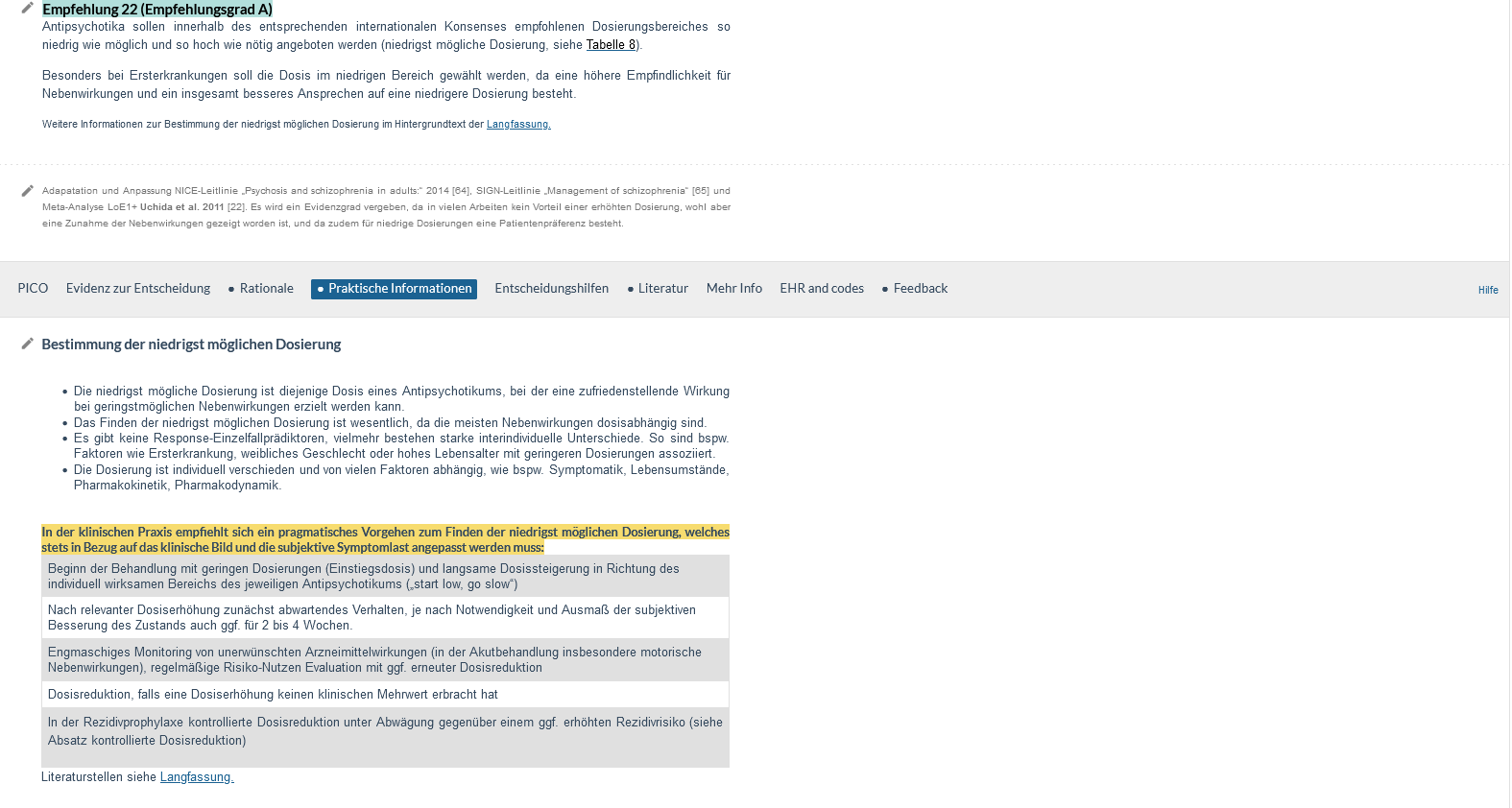
*From “www.app.magicapp.org S3-Leitlinie Schizophrenie* (2023) [cited 2023 May 18]. Not publicly available”.

**Figure 5: Decision aids for shared decision-making in MAGICapp**


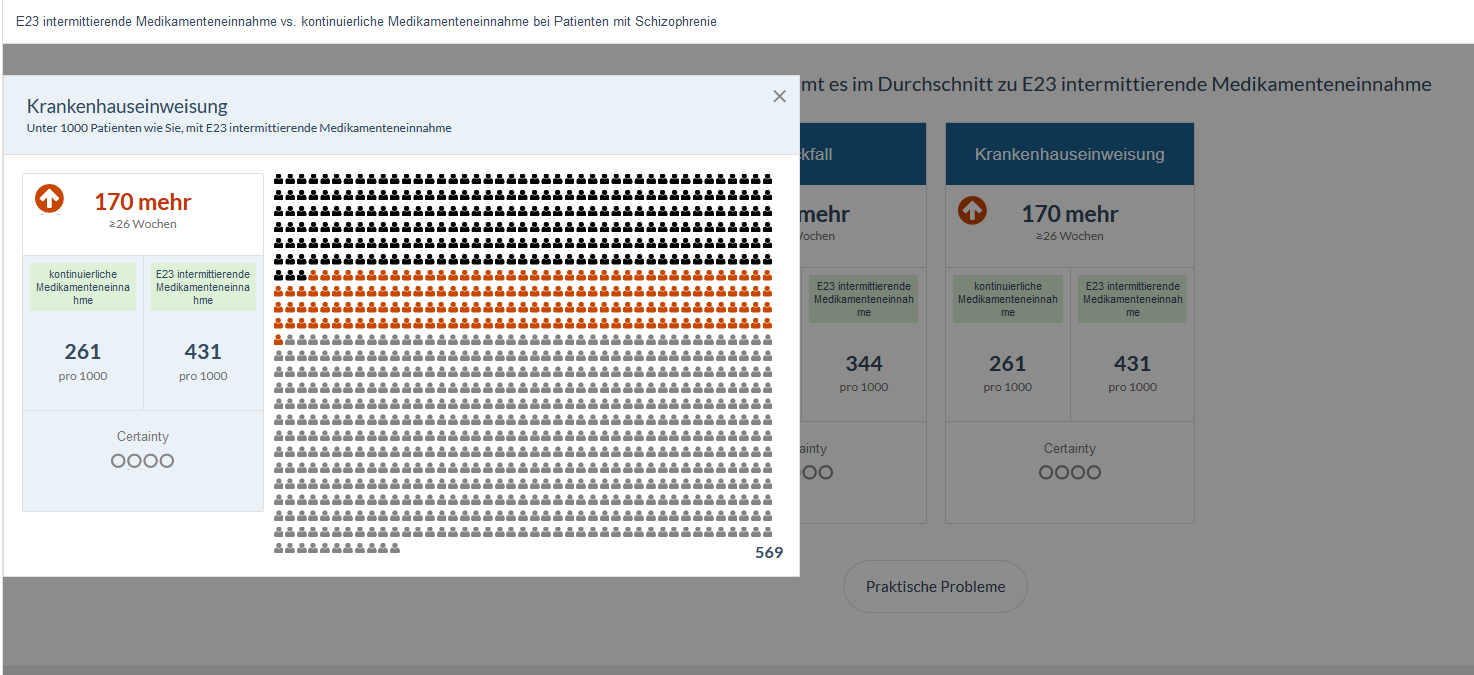


*From “www.app.magicapp.org S3-Leitlinie Schizophrenie* (2023) [cited 2023 May 18]. Not publicly available”.

**Figure 6: Features of MAGICapp**


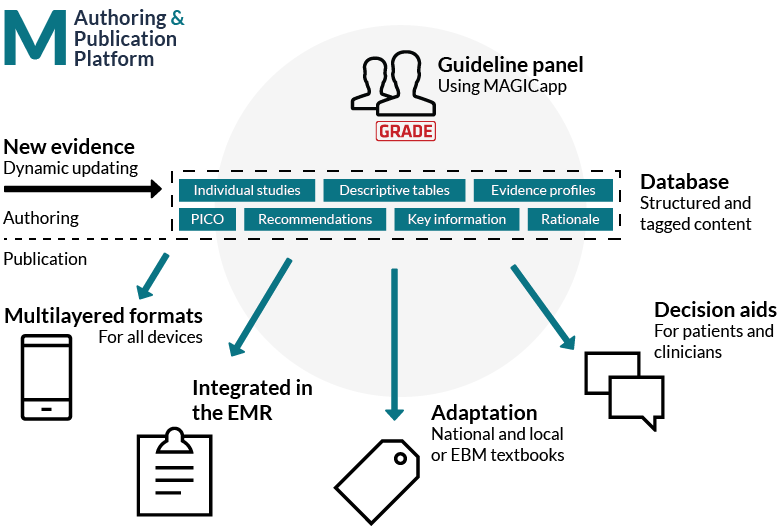


*From “MAGICevidence website* (2023) [cited 2023 May 18]. Available from: https://magicevidence.magicapp.org/magicapp/”
