# Supplementary material for: The impact of a digital guideline version on schizophrenia guideline knowledge: results from a multicenter cluster-randomized controlled trial
Source: BMC Med. 2024 Jul 29;22:311. doi: 10.1186/s12916-024-03533-6 (PMC11287881; doi:10.1186/s12916-024-03533-6)
Supplement: Supplementary file 2 — Additional file 2: Table 1. Questionnaire assessing attitudes towards and use of the German schizophrenia guideline. Table 2. Primary and secondary outcomes CG vs. IG (at T0 and T1). Table 3. Primary and secondary outcomes T0 vs. T1 (for CG and IG). Table 4. Demographic/occupational characteristics for primary outcome (success vs. failed) among entire sample. Table 5. Secondary outcomes print format vs. MAGICapp (exclusively in the IG) at T1. [file 12916_2024_3533_MOESM2_ESM.docx]

**Table 1: Questionnaire assessing attitudes towards and use of the German schizophrenia guideline**

|  | Completely disagree | Rather disagree | Neither disagree nor agree | Tend to agree | Agree completely |
| --- | --- | --- | --- | --- | --- |
| I can very well imagine using the guideline on a regular basis. |  |  |  |  |  |
| I find the guideline unnecessarily complex. |  |  |  |  |  |
| I find the guideline easy to use. |  |  |  |  |  |
| I think that I would need support (contact person) to use the guideline. |  |  |  |  |  |
| I feel that the various features of the guideline are well integrated. |  |  |  |  |  |
| I find that there are too many inconsistencies in the guideline. |  |  |  |  |  |
| I imagine that most people will learn to use the guideline quickly. |  |  |  |  |  |
| I find it very inconvenient to use. |  |  |  |  |  |
| I felt very confident using the guideline in print format. |  |  |  |  |  |
| I had to learn a lot of things before I could work with the guideline in print format. |  |  |  |  |  |

**Table 2: Primary and secondary outcomes CG vs IG (at T0 and T1)**

|  | **T0** | | | | | | | **T1** | | | | | | |
| --- | --- | --- | --- | --- | --- | --- | --- | --- | --- | --- | --- | --- | --- | --- |
|  | **CG** | | **IG** | | **CG vs. IG** | | | **CG** | | **IG** | | **CG vs. IG** | | |
| **Efficacy Outcomes** |  | | | | | | | | | | | | | |
|  | **N** | **%** | **N** | **%** | **Χ²** | **df** | **p_a_** | **N** | **%** | **N** | **%** | **Χ²** | **df** | **p_a_** |
| Primary Outcome | 110 | 43.6 | 101 | 52.5 | 1.65 | 1 | 0.199 | 67 | 58.2 | 52 | 63.5 | 0.34 | 1 | 0.561 |
|  | **N** | **%** | **N** | **%** | **U** | **Z** | **p_b_** | **N** | **%** | **N** | **%** | **U** | **Z** | **p_b_** |
| Percentage of correctly answered questions | 114 | 68.59 | 103 | 68.27 | 5843.0 | -0.06 | 0.952 | 68 | 72.38 | 52 | 72.12 | 1737.5 | -0.16 | 0.871 |
|  |  | | | | | | | | | | | | | |
|  | **N** | **M ± SD** | **N** | **M ± SD** | **U** | **Z** | **p_b_** | **N** | **M ± SD** | **N** | **M ± SD** | **U** | **Z** | **p_b_** |
| **Use of guideline** |  |  |  |  |  |  |  |  |  |  |  |  |  |  |
| Total (sum use of guideline) | 114 | 12.35 ± 2.37 | 103 | 11.76 ± 2.26 | 5124.5 | -1.63 | 0.103 | 68 | 12.47 ± 2.78 | 52 | 11.87 ± 3.27 | 1554.5 | -1.14 | 0.256 |
| How often did you refer to the guideline for clinical work within the past 6 months? ^a^ | 114 | 1.98  ± 0.84 | 103 | 1.95  ± 0.85 | 5743.0 | -0.30 | 0.768 | 68 | 2.35  ± 0.82 | 52 | 2.04  ± 0.86 | 1377.0 | -2.21 | 0.027* |
| I feel confident using the guideline in print format. ^b^ | 114 | 2.94  ± 0.94 | 103 | 2.62  ± 0.97 | 4896.0 | -2.22 | 0.026* | 68 | 3.31  ± 0.97 | 52 | 3.08  ± 1.20 | 1585.0 | -1.02 | 0.307 |
| I consider the guideline in print format to be useful. ^b^ | 114 | 4.25  ± 0.69 | 103 | 4.10  ± 0.60 | 5115.0 | -1.83 | 0.068 | 68 | 3.65  ± 0.91 | 52 | 3.69  ± 0.97 | 1662.5 | -0.59 | 0.553 |
| The guideline in print format facilitates my daily work. ^b^ | 114 | 3.18  ± 0.73 | 103 | 3.09  ± 0.83 | 5664.0 | -0.50 | 0.615 | 68 | 3.16  ± 1.09 | 52 | 3.06  ± 1.15 | 1684.0 | -0.46 | 0.645 |
| **User-friendliness of guideline in print format** |  | | | | | | | | | | | | | |
| Total (sum user-friendliness) | 114 | 22.25  ± 5.40 | 103 | 21.25  ± 4.11 | 5006.5 | -1.88 | 0.061 | 68 | 23.22  ± 6.09 | 52 | 22.67  ± 6.94 | 1675.5 | -0.49 | 0.624 |
| I can well imagine using the guideline in print format on a regular basis. ^d^ | 114 | 2.82  ± 0.81 | 103 | 2.68  ± 0.84 | 5291.5 | -1.38 | 0.166 | 68 | 2.38  ± 0.96 | 52 | 2.27  ± 1.12 | 1670.5 | -0.55 | 0.585 |
| I find the guideline in print format unnecessarily complex. ^e^ | 114 | 2.06  ± 1.04 | 103 | 1.93  ± 0.91 | 5451.5 | -0.95 | 0.343 | 68 | 2.09  ± 0.97 | 52 | 2.08  ± 1.01 | 1726.0 | -0.23 | 0.816 |
| I find the guideline easy to use in print format. ^d^ | 114 | 2.04  ± 0.79 | 103 | 1.84  ± 0.78 | 5063.0 | -1.87 | 0.062 | 68 | 2.21  ± 0.86 | 52 | 2.17  ± 0.96 | 1733.5 | -0.20 | 0.845 |
| I think that I would need support (contact person) to use the guideline in print format. ^e^ | 114 | 2.29  ± 1.05 | 103 | 2.19  ± 1.01 | 5559.5 | -0.71 | 0.480 | 68 | 2.75  ± 1.07 | 52 | 2.67  ± 1.08 | 1690.5 | -0.43 | 0.668 |
| I think that the various functions of the guideline are well integrated in the print format. ^d^ | 114 | 2.17  ± 0.64 | 103 | 2.13  ± 0.70 | 5720.5 | -0.37 | 0.712 | 68 | 2.09  ± 0.77 | 52 | 1.98  ± 0.83 | 1612.0 | -0.88 | 0.378 |
| I find that there are too many inconsistencies in the guidelines in print format. ^e^ | 114 | 2.31  ± 0.65 | 103 | 2.32  ± 0.63 | 5814.0 | -0.14 | 0.888 | 68 | 2.51  ± 0.74 | 52 | 2.44  ± 0.73 | 1667.0 | -0.59 | 0.557 |
| I can imagine that most people quickly learn to work with the guideline in print format. ^d^ | 114 | 2.03  ± 0.88 | 103 | 1.97  ± 0.81 | 5558.0 | -0.72 | 0.474 | 68 | 2.24  ± 0.81 | 52 | 2.08  ± 1.01 | 1619.0 | -0.83 | 0.405 |
| I find the handling very complicated. ^e^ | 114 | 2.03  ± 0.90 | 103 | 1.89  ± 0.75 | 5305.5 | -1.31 | 0.190 | 68 | 2.12  ± 0.89 | 52 | 2.17  ± 1.12 | 1730.5 | -0.21 | 0.835 |
| I felt very confident using the guideline in print format. ^d^ | 114 | 2.10  ± 0.87 | 103 | 2.09  ± 0.73 | 5787.0 | -0.20 | 0.843 | 68 | 2.31  ± 0.80 | 52 | 2.19  ± 0.99 | 1662.0 | -0.60 | 0.549 |
| I had to learn a lot of things before I could work with the guideline in print format. ^e^ | 114 | 2.41  ± 0.97 | 103 | 2.20  ± 0.82 | 5127.0 | -1.71 | 0.087 | 68 | 2.53  ± 0.97 | 52 | 2.62  ± 1.11 | 1641.5 | -0.70 | 0.482 |
| **Shared Decision Making** |  | | | | | | | | | | | | | |
| Total (sum shared decision making) | 114 | 42.21  ± 6.40 | 103 | 41.03  ± 7.74 | 5430.0 | -0.96 | 0.339 | 68 | 42.79  ± 5.36 | 52 | 42.56  ± 7.54 | 1762.5 | -0.03 | 0.977 |
| I made clear to my patient that a decision needs to be made. ^f^ | 114 | 4.44  ± 1.07 | 103 | 4.38  ± 1.19 | 5862.5 | -0.02 | 0.985 | 68 | 4.65  ± 0.82 | 52 | 4.65  ± 1.14 | 1712.0 | -0.32 | 0.752 |
| I wanted to know exactly from my patient how he/she wants to be involved in making the decision. ^f^ | 114 | 4.32  ± 1.01 | 103 | 4.21  ± 1.13 | 5667.5 | -0.46 | 0.646 | 68 | 4.37  ± 0.98 | 52 | 4.33  ± 1.02 | 1723.5 | -0.25 | 0.804 |
| I told my patient that there are different options for treating his/her medical condition. ^f^ | 114 | 4.95  ± 0.83 | 103 | 4.84  ± 1.04 | 5753.5 | -0.27 | 0.785 | 68 | 5.07  ± 0.70 | 52 | 4.94  ± 0.94 | 1673.0 | -0.55 | 0.581 |
| I precisely explained the advantages and disadvantages of the treatment options to my patient. ^f^ | 114 | 4.70  ± 0.98 | 103 | 4.53  ± 1.12 | 5472.5 | -0.92 | 0.360 | 68 | 4.74  ± 0.77 | 52 | 4.75  ± 1.06 | 1643.0 | -0.71 | 0.480 |
| I helped my patient understand all the information. ^f^ | 114 | 4.97  ± 0.85 | 103 | 4.81  ± 0.95 | 5380.5 | -1.16 | 0.248 | 68 | 4.88  ± 0.74 | 52 | 4.94  ± 0.87 | 1633.0 | -0.78 | 0.436 |
| I asked my patient which treatment option he/she prefers. ^f^ | 114 | 4.93  ± 0.96 | 103 | 4.79  ± 1.09 | 5544.5 | -0.75 | 0.454 | 68 | 4.94  ± 0.86 | 52 | 4.96  ± 1.01 | 1697.0 | -0.40 | 0.690 |
| My patient and I thoroughly weighed the different treatment options. ^f^ | 114 | 4.50  ± 0.95 | 103 | 4.32  ± 1.09 | 5377.5 | -1.13 | 0.259 | 68 | 4.56  ± 0.85 | 52 | 4.58  ± 1.13 | 1687.5 | -0.46 | 0.648 |
| My patient and I selected a treatment option together. ^f^ | 114 | 4.58  ± 0.99 | 103 | 4.49  ± 1.10 | 5681.0 | -0.43 | 0.664 | 68 | 4.68  ± 0.76 | 52 | 4.58  ± 0.87 | 1643.0 | -0.72 | 0.469 |
| My patient and I reached an agreement on how to proceed. ^f^ | 114 | 4.82  ± 0.94 | 103 | 4.66  ± 1.14 | 5568.5 | -0.69 | 0.492 | 68 | 4.91  ± 0.84 | 52 | 4.83  ± 1.10 | 1754.5 | -0.08 | 0.939 |
| **Provider Decision Process** |  | | | | | | | | | | | | | |
| Total (sum provider decision process) | 114 | 33.32  ± 5.07 | 103 | 33.33  ± 6.12 | 5818.0 | -0.12 | 0.908 | 68 | 41.62  ± 4.72 | 52 | 40.13  ± 5.78 | 1558.5 | -1.11 | 0.266 |
| The decision was hard to make. ^c^ | 114 | 3.14  ± 0.83 | 103 | 3.06  ± 0.83 | 5523.5 | 0.80 | 0.421 | 68 | 3.07  ± 0.76 | 52 | 3.00  ± 0.95 | 1673.5 | -0.53 | 0.597 |
| I was unsure what treatment would really be best for this patient. ^c^ | 114 | 2.89  ± 0.94 | 103 | 2.94  ± 0.97 | 5739.0 | -0.30 | 0.762 | 68 | 3.49  ± 0.82 | 52 | 3.31  ± 0.90 | 1597.0 | -1.00 | 0.318 |
| It was clear what treatment would be best for this patient. ^b^ | 114 | 2.75  ± 0.86 | 103 | 2.90  ± 0.82 | 5316.0 | -1.28 | 0.201 | 68 | 3.50  ± 0.78 | 52 | 3.15  ± 0.89 | 1402.0 | -2.10 | 0.036* |
| When making the decision, I felt I did not know enough about the treatment alternatives, although the information is available in the literature. ^c^ | 114 | 2.83  ± 1.02 | 103 | 2.78  ± 1.00 | 5666.5 | -0.47 | 0.643 | 68 | 3.65  ± 0.89 | 52 | 3.33  ± 1.06 | 1462.5 | -1.74 | 0.082 |
| I had trouble making the decision because important information is either unknown or not readily available in the literature. ^c^ | 114 | 2.55  ± 0.96 | 103 | 2.63  ± 1.04 | 5666.0 | -0.47 | 0.642 | 68 | 3.74  ± 0.87 | 52 | 3.40  ± 1.09 | 1492.0 | -1.57 | 0.116 |
| When I made the decision, it was hard to decide if the benefits of the available treatments were more important than the risks or vice versa. ^c^ | 114 | 2.88  ± 0.95 | 103 | 2.81  ± 0.98 | 5648.5 | -0.51 | 0.613 | 68 | 3.19  ± 0.82 | 52 | 3.44  ± 0.98 | 1477.0 | -1.64 | 0.101 |
| It was easy to identify all of the considerations that affect the decision. ^b^ | 114 | 3.25  ± 0.84 | 103 | 3.17  ± 0.88 | 5581.5 | -0.67 | 0.506 | 68 | 2.99  ± 0.78 | 52 | 2.94  ± 0.78 | 1724.0 | -0.25 | 0.803 |
| I fully understand the patient´s views regarding the important issues in making this decision. ^b^ | 114 | 1.97  ± 0.78 | 103 | 1.97  ± 0.75 | 5799.0 | -0.17 | 0.864 | 68 | 4.09  ± 0.81 | 52 | 3.96  ± 0.77 | 1565.5 | -1.18 | 0.239 |
| I believe that the patient fully understands the risks and benefits of the treatment I prescribed. ^b^ | 114 | 3.25  ± 0.82 | 103 | 3.17  ± 0.86 | 5553.5 | -0.73 | 0.465 | 68 | 3.12  ± 0.82 | 52 | 3.04  ± 0.79 | 1626.5 | -0.81 | 0.419 |
| I believe that the patient will comply with the treatment regimen I prescribed. ^b^ | 114 | 2.92  ± 0.69 | 103 | 2.92  ± 0.74 | 5846.0 | -0.06 | 0.952 | 68 | 3.28  ± 0.73 | 52 | 3.15  ± 0.78 | 1630.5 | -0.79 | 0.428 |
| I am satisfied with the decision that was made. ^b^ | 114 | 2.35  ± 0.61 | 103 | 2.48  ± 0.59 | 5202.5 | -1.67 | 0.094 | 68 | 3.69  ± 0.73 | 52 | 3.69  ± 0.70 | 1718.5 | -0.31 | 0.756 |
| I am satisfied that the process used to make the decision was as good as it could be. ^b^ | 114 | 2.55  ± 0.82 | 103 | 2.51  ± 0.86 | 5682.0 | -0.45 | 0.656 | 68 | 3.82  ± 0.83 | 52 | 3.71  ± 0.91 | 1683.0 | -0.50 | 0.615 |
| **eHealth literacy (personal health-related use of the Internet in everyday life)** |  | | | | | | | | | | | | | |
| Total (sum eHealth literacy health-related - everxday life) | 114 | 32.58  ± 4.34 | 103 | 30.47  ± 5.99 | 4748.0 | -2.44 | 0.015* | 68 | 32.19  ± 4.71 | 52 | 31.38  ± 4.93 | 1513.0 | -1.36 | 0.175 |
| I know what health resources are available on the Internet.^b^ | 114 | 3.83  ± 0.80 | 103 | 3.68  ± 0.92 | 5471.5 | -0.95 | 0.342 | 68 | 3.94  ± 0.69 | 52 | 3.83  ± 0.76 | 1622.0 | -0.89 | 0.371 |
| I know where to find helpful health resources on the Internet. ^b^ | 114 | 4.03  ± 0.76 | 103 | 3.76  ± 0.87 | 4965.0 | -2.16 | 0.031* | 68 | 4.07  ± 0.68 | 52 | 3.96  ± 0.71 | 1608.0 | -1.00 | 0.316 |
| I know how to find helpful health resources on the Internet. ^b^ | 114 | 4.09  ± 0.71 | 103 | 3.89  ± 0.87 | 5262.0 | -1.48 | 0.139 | 68 | 4.03  ± 0.69 | 52 | 4.04  ± 0.69 | 1761.0 | -0.04 | 0.965 |
| I know how to use the Internet to answer my questions about health. ^b^ | 114 | 4.18  ± 0.76 | 103 | 3.83  ± 0.91 | 4657.5 | -2.91 | 0.004* | 68 | 4.06  ± 0.83 | 52 | 4.10  ± 0.77 | 1743.0 | -0.15 | 0.883 |
| I know how to use the health information I find on the Internet to help me. ^b^ | 114 | 4.24  ± 0.74 | 103 | 3.93  ± 0.80 | 4606.5 | -3.02 | 0.003* | 68 | 4.16  ± 0.68 | 52 | 4.04  ± 0.74 | 1607.5 | -0.98 | 0.328 |
| I have the skills I need to evaluate the health resources I find on the Internet. ^b^ | 114 | 4.37  ± 0.63 | 103 | 4.05  ± 0.89 | 4763.0 | -2.68 | 0.007* | 68 | 4.18  ± 0.73 | 52 | 4.02  ± 0.78 | 1588.5 | -1.06 | 0.291 |
| I can tell high quality health resources from low quality health resources on the Internet. ^b^ | 114 | 4.24  ± 0.68 | 103 | 3.96  ± 0.91 | 5011.0 | -2.08 | 0.038* | 68 | 4.06  ± 0.69 | 52 | 3.87  ± 0.66 | 1519.0 | -1.52 | 0.129 |
| I feel confident in using information from the Internet to make health decisions. ^b^ | 114 | 3.61  ± 0.77 | 103 | 3.36  ± 0.90 | 4941.5 | -2-15 | 0.031* | 68 | 3.69  ± 0.89 | 52 | 3.54  ± 0.83 | 1563.5 | -1.16 | 0.247 |
| **eHealth literacy (health-related use of the Internet for work purposes)** |  | | | | | | | | | | | | | |
| Total (sum eHealth literacy health-related – work purposes) | 114 | 32.22  ± 4.89 | 103 | 30.01  ± 6.13 | 4590.0 | -2.78 | 0.005* | 68 | 32.22  ± 4.99 | 52 | 31.31  ± 5.62 | 1614.5 | -0.82 | 0.411 |
| I know what health resources are available on the Internet. ^b^ | 114 | 3.86  ± 0.83 | 103 | 3.59  ± 0.95 | 5047.0 | -1.92 | 0.055 | 68 | 3.87  ± 0.81 | 52 | 3.77  ± 0.73 | 1607.5 | -0.97 | 0.334 |
| I know where to find helpful health resources on the Internet. ^b^ | 114 | 3.98  ± 0.85 | 103 | 3.68  ± 0.89 | 4824.0 | -2.44 | 0.015* | 68 | 4.01  ± 0.78 | 52 | 3.88  ± 0.76 | 1590.0 | -1.06 | 0.288 |
| I know how to find helpful health resources on the Internet. ^b^ | 114 | 4.00  ± 0.80 | 103 | 3.80  ± 0.88 | 5166.0 | -1.68 | 0.093 | 68 | 4.01  ± 0.82 | 52 | 3.94  ± 0.75 | 1673.0 | -0.57 | 0.571 |
| I know how to use the Internet to answer my questions about health. ^b^ | 114 | 4.00  ± 0.79 | 103 | 3.73  ± 0.88 | 4833.0 | -2.44 | 0.015* | 68 | 4.07  ± 0.82 | 52 | 4.00  ± 0.82 | 1691.0 | -0.45 | 0.651 |
| I know how to use the health information I find on the Internet to help me. ^b^ | 114 | 4.14  ± 0.75 | 103 | 3.86  ± 0.88 | 4882.5 | -2.34 | 0.020* | 68 | 4.16  ± 0.64 | 52 | 4.02  ± 0.83 | 1649.5 | -0.72 | 0.470 |
| I have the skills I need to evaluate the health resources I find on the Internet. ^b^ | 114 | 4.32  ± 0.68 | 103 | 4.01  ± 0.87 | 4781.5 | -2.61 | 0.009* | 68 | 4.16  ± 0.77 | 52 | 4.02  ± 0.85 | 1620.0 | -0.87 | 0.384 |
| I can tell high quality health resources from low quality health resources on the Internet. ^b^ | 114 | 4.25  ± 0.70 | 103 | 3.94  ± 0.85 | 4784.5 | -2.61 | 0.009* | 68 | 4.10  ± 0.67 | 52 | 4.00  ± 0.86 | 1705.5 | -0.37 | 0.713 |
| I feel confident in using information from the Internet to make health decisions. ^b^ | 114 | 3.68  ± 0.87 | 103 | 3.40  ± 0.92 | 4869.0 | -2.30 | 0.021* | 68 | 3.82  ± 0.81 | 52 | 3.67  ± 0.83 | 1598.5 | -1.00 | 0.317 |

CG: Control Group, IG: Intervention Group, T0: Baseline survey, T1: Post-intervention survey, N: number (group size), M: mean, SD: Standard deviation, Χ²: Chi²-statistic, df: Degrees of freedom, p_a_: p-value from Chi-square test, U: Mann-Whitney U, Z: Z-statistic, p_b_: p-value from Mann-Whitney U-test, ^a^: 5-point Likert scale: 1=never, 5=daily, ^b^: 5-point Likert scale: 1=strongly disagree, 5=strongly agree, ^c^: 5-point Likert scale: 1=strongly agree, 5=strongly disagree, ^d^: 5-point Likert scale: 0=strongly disagree, 4=strongly agree, ^e^: 5-point Likert scale: 0=strongly agree, 4=strongly disagree, ^f^: 6-point Likert scale: 1=applies not at all, 6=applies strongly, * p < 0.005

**Table 3: Primary and secondary outcomes T0 vs T1 (for CG and IG)**

|  | **Control group T0 vs. T1** | | | **Experimental group T0 vs. T1** | | |
| --- | --- | --- | --- | --- | --- | --- |
| **Efficacy Outcomes** |  | | | | | |
|  | **Χ²** | **df** | **p_a_** | **Χ²** | **df** | **p_a_** |
| Primary Outcome (≥65% correct, all index q. correct ? (yes / no)) | 3.54 | 1 | 0.060 | 1.68 | 1 | 0.194 |
|  | **Z** | | **p_c_** | **Z** | | **p_c_** |
| Percentage of correct answers | -2.20 | | 0.028* | -1.35 | | 0.177 |
| **Use of guideline** |  | | | | | |
| Total (sum use of guideline) | -0.43 | | 0.668 | -0.20 | | 0.842 |
| How often did you refer to the guideline for clinical work within the past 6 months? | -3.31 | | 0.001* | -0.36 | | 0.717 |
| I feel confident using the guideline in print format. | -2.48 | | 0.013* | -2.85 | | 0.004* |
| I consider the guideline in print format to be useful. | -4.41 | | <0.001* | -2.96 | | 0.003* |
| The guideline in print format facilitates my daily work. | -0.99 | | 0.324 | -1.63 | | 0.103 |
| **User-friendliness of guideline in print format** |  | | | | | |
| Total (sum user-friendliness) | -0.76 | | 0.447 | -1.24 | | 0.216 |
| I can well imagine using the guideline in print format on a regular basis. | -3.53 | | <0.001* | -2.45 | | 0.014* |
| I find the guideline in print format unnecessarily complex. | -0.03 | | 0.977 | -0.99 | | 0.323 |
| I find the guideline easy to use in print format. | -0.70 | | 0.482 | -2.78 | | 0.005* |
| I think that I would need support (contact person) to use the guideline in print format. | -2.40 | | 0.016* | -3.00 | | 0.003* |
| I think that the various functions of the guideline are well integrated in the print format. | -1.91 | | 0.057 | -1.20 | | 0.231 |
| I find that there are too many inconsistencies in the guidelines in print format. | -1.07 | | 0.286 | -0.06 | | 0.952 |
| I can imagine that most people quickly learn to work with the guideline in print format. | -1.00 | | 0.316 | -0.67 | | 0.503 |
| I find the handling very complicated in print format. | -0.05 | | 0.960 | -1.03 | | 0.302 |
| I felt very confident using the guideline in print format. | -0.97 | | 0.330 | -0.21 | | 0.831 |
| I had to learn a lot of things before I could work with the guideline in print format. | -0.46 | | 0.647 | -2.59 | | 0.009* |
| **Shared Decision Making** |  | | | | | |
| Total (sum shared decision making) | -1.61 | | 0.108 | -3.17 | | 0.002* |
| I made clear to my patient that a decision needs to be made. | -1.42 | | 0.156 | -1.64 | | 0.101 |
| I wanted to know exactly from my patient how he/she wants to be involved in making the decision. | -0.77 | | 0.443 | -2.06 | | 0.040* |
| I told my patient that there are different options for treating his/her medical condition. | -1.64 | | 0.100 | --0.87 | | 0.386 |
| I precisely explained the advantages and disadvantages of the treatment options to my patient. | -0.16 | | 0.874 | -1.95 | | 0.051 |
| I helped my patient understand all the information. | -0.52 | | 0.603 | -1.23 | | 0.221 |
| I asked my patient which treatment option he/she prefers. | -1.00 | | 0.317 | -1.74 | | 0.082 |
| My patient and I thoroughly weighed the different treatment options. | -0.65 | | 0.517 | -2.44 | | 0.015* |
| My patient and I selected a treatment option together. | -1.35 | | 0.176 | -1.73 | | 0.085 |
| My patient and I reached an agreement on how to proceed. | -0.68 | | 0.496 | -2.62 | | 0.009* |
| **Provider Decision Process** |  | | | | | |
| Total (sum provider decision process) | -6.21 | | <0.001* | -3.65 | | <0.001* |
| The decision was hard to make. | -0.44 | | 0.661 | -0.07 | | 0.948 |
| I was unsure what treatment would really be best for this patient. | -3.51 | | <0.001* | -1.37 | | 0.172 |
| It was clear what treatment would be best for this patient. | -4.14 | | <0.001* | -0.80 | | 0.424 |
| When making the decision, I felt I did not know enough about the treatment alternatives, although the information is available in the literature. | -3.55 | | <0.001* | -1.96 | | 0.050 |
| I had trouble making the decision because important information is either unknown or not readily available in the literature. | -5.01 | | <0.001* | -3.02 | | 0.003* |
| When I made the decision, it was hard to decide if the benefits of the available treatments were more important than the risks or vice versa. | -1.64 | | 0.101 | -2.47 | | 0.014* |
| It was easy to identify all of the considerations that affect the decision. | -1.10 | | 0.270 | -1.79 | | 0.073 |
| I fully understand the patient´s views regarding the important issues in making this decision. | -6.93 | | <0.001* | -5.93 | | <0.001* |
| I believe that the patient fully understands the risks and benefits of the treatment I prescribed. | -0.45 | | 0.650 | -1.01 | | 0.315 |
| I believe that the patient will comply with the treatment regimen I prescribed. | -2.91 | | 0.004* | -1.08 | | 0.282 |
| I am satisfied with the decision that was made. | -6.61 | | <0.001* | -5.33 | | <0.001* |
| I am satisfied that the process used to make the decision was as good as it could be. | -6.07 | | <0.001* | -4.22 | | <0.001* |
| **eHealth literacy (personal health-related use of the Internet in everyday life)** |  | | | | | |
| Total (sum eHealth literacy health-related – everyday-life) | -0.28 | | 0.782 | -1.13 | | 0.259 |
| I know what health resources are available on the Internet. | -1.12 | | 0.265 | -1.24 | | 0.217 |
| I know where to find helpful health resources on the Internet. | -0.84 | | 0.400 | -1.37 | | 0.170 |
| I know how to find helpful health resources on the Internet. | -0.67 | | 0.502 | -0.85 | | 0.396 |
| I know how to use the Internet to answer my questions about health. | -0.33 | | 0.741 | -1.43 | | 0.154 |
| I know how to use the health information I find on the Internet to help me. | -1.00 | | 0.318 | -0.51 | | 0.610 |
| I have the skills I need to evaluate the health resources I find on the Internet. | -2.16 | | 0.031 | -0.45 | | 0.655 |
| I can tell high quality health resources from low quality health resources on the Internet. | -2.40 | | 0.016 | -0.37 | | 0.715 |
| I feel confident in using information from the Internet to make health decisions. | -1.64 | | 0.100 | -1.29 | | 0.196 |
| **eHealth literacy (health-related use of the Internet for work purposes)** |  | | | | | |
| Total (sum eHealth literacy health-related – work purposes) | -0.68 | | 0.497 | -1.76 | | 0.079 |
| I know what health resources are available on the Internet. | -0.19 | | 0.850 | -1.58 | | 0.114 |
| I know where to find helpful health resources on the Internet. | -0.70 | | 0.486 | -1.53 | | 0.118 |
| I know how to find helpful health resources on the Internet. | -0.27 | | 0.788 | -1.29 | | 0.196 |
| I know how to use the Internet to answer my questions about health. | -1.04 | | 0.299 | -2.55 | | 0.011* |
| I know how to use the health information I find on the Internet to help me. | -0.32 | | 0.747 | -0.79 | | 0.429 |
| I have the skills I need to evaluate the health resources I find on the Internet. | -1.77 | | 0.076 | -0.28 | | 0.783 |
| I can tell high quality health resources from low quality health resources on the Internet. | -2.20 | | 0.028* | -1.01 | | 0.311 |
| I feel confident in using information from the Internet to make health decisions. | -1.95 | | 0.052 | -2.12 | | 0.034* |

T0: Baseline survey, T1: Post-intervention survey, CG: Control Group, IG: Intervention Group, Χ²: Chi²-statistic, df: Degrees of freedom, p_a_: p-value from Chi-square test, Z: Z-statistic; p_c_: p-value from Wilcoxon test, q. question, * p < 0.005

**Table 4: Demographic/occupational characteristics for primary outcome (success vs. failed) among entire sample**

|  | **Primary Outcome** (at least 65% correct answers and all index questions answered correctly? (yes / no)) | | | | | | | | | | | |
| --- | --- | --- | --- | --- | --- | --- | --- | --- | --- | --- | --- | --- |
|  | **T0** | | | | | | **T1** | | | | | |
|  | **Success** | | **Failed** | | **Success vs. failed** | | **Success** | | **Failed** | | **Success vs. failed** | |
|  | N | M  ± SD | N | M  ± SD | Z | p_b_ | N | M  ± SD | N | M  ± SD | Z | p_b_ |
| **Age** | 101 | 39.17  ± 9.96 | 110 | 35.45  ± 8.54 | -2.73 | 0.006* | 72 | 40.39  ± 9.59 | 47 | 34.13  ± 6.49 | -3.71 | <0.001* |
| **Work experience** | 96 | 10.56  ± 8.63 | 102 | 6.28  ± 5.91 | -3.81 | <0.001* | 66 | 11.47  ± 9.00 | 47 | 5.64  ± 5.25 | -3.71 | <0.001* |
|  | **T0** | | | | | | **T1** | | | | | |
|  | **Success** | | **Failed** | | **Success vs. failed** | | **Success** | | **Failed** | | **Success vs. failed** | |
|  | **N** | **%** | N | **%** | **Value** | **p_d_** | **N** | **%** |  |  | **Value** | **p_d_** |
| **Profession** |  |  |  |  | 49.89 | <0.001* |  |  |  |  | 33.28 | <0.001* |
| *Psychologist/psychotherapist* |  |  |  |  |  |  |  |  |  |  |  |  |
| Psychotherapist | 5 | 20.0 | 20 | 80.0 |  |  | 6 | 37.5 | 10 | 62.5 |  |  |
| Psychotherapist in training | 10 | 29.4 | 24 | 70.6 |  |  | 5 | 33.3 | 10 | 66.7 |  |  |
| Psychologist | 3 | 15.0 | 17 | 85.0 |  |  | 1 | 11.1 | 8 | 88.9 |  |  |
| *Physicians* |  |  |  |  |  |  |  |  |  |  |  |  |
| Specialist in psychiatry and psychotherapy | 44 | 80.0 | 11 | 20.0 |  |  | 34 | 91.9 | 3 | 8.1 |  |  |
| Assistant physician in psychiatry and psychotherapy | 35 | 52.2 | 32 | 47.8 |  |  | 25 | 61.0 | 16 | 39.0 |  |  |
| Assistant physician in psychosomatic medicine and psychotherapy | 2 | 66.7 | 1 | 33.3 |  |  | - | - | - | - |  |  |
| Specialist in neurology | 1 | 25.0 | 3 | 75.0 |  |  | - | - | - | - |  |  |
| Assistant physician in neurology | 0 | 0.0 | 1 | 100.0 |  |  | - | - | - | - |  |  |
| Assistant physician for general medicine | 0 | 0.0 | 1 | 100.0 |  |  | - | - | - | - |  |  |
| Specialist physician in another field | 1 | 100.0 | 0 | 0.0 |  |  | 1 | 100.0 | 0 | 0.0 |  |  |
|  | **Success** | | **Failed** | |  | | **Success** | | **Failed** | |  | |
|  | **N** | **%** | N | **%** | **Χ²** | **p_a_** | **N** | **%** |  |  | **Χ²** | **p_a_** |
|  |  |  |  |  |  |  |  |  |  |  |  |  |
| **Participation in expert boards** |  |  |  |  |  |  |  |  |  |  | 8.87 | 0.003* |
| Participated at least once |  |  |  |  |  |  | 49 | 72.1 | 19 | 27.9 |  |  |
| Never participated |  |  |  |  |  |  | 23 | 45.1 | 28 | 54.9 |  |  |
| **Participation in training session** |  |  |  |  |  |  |  |  |  |  | 0.27 | 0.604 |
| Participated/viewed recording at least once |  |  |  |  |  |  | 58 | 61.7 | 36 | 38.3 |  |  |
| Never participated/viewed recording |  |  |  |  |  |  | 14 | 56.0 | 11 | 44.0 |  |  |

T0: Baseline survey, T1: Post-intervention survey, Success: at least 65% correct answers and all index questions answered correctly, Failed: less than 65% answered correctly or at least one index question answered wrong, N: number (group size), M: mean, SD: Standard deviation, Χ²: Chi²-statistic; df: Degrees of freedom, p_a_: p-value from Chi-square test, p_b_: p-value from Mann-Whitney U-test, p_d_: p-value from Exact Fisher-Freeman-Halton test; * p < 0.005

**Table 5: Secondary outcomes print format vs MAGICapp (exclusively in the IG) at T1**

|  | **Print format** | | **MAGICapp** | | **Print format vs. MAGICapp** | |
| --- | --- | --- | --- | --- | --- | --- |
|  | **N** | **M**  **± SD** | **N** | **M**  **± SD** | **Z** | **p_b_** |
| How often did you use the guideline for clinical work within the past 6 months?^a^ | 52 | 2.04  ± 0.86 | 51 | 1.57  ± 0.72 | -3.16 | 0.002* |
| **Use of guideline** |  |  |  |  |  |  |
| Total (sum use of guideline) | 52 | 13.83  ± 3.86 | 51 | 12.67  ± 3.59 | -1.45 | 0.148 |
| How often did you refer to the guideline within the past 6 months? ^a^ | 52 | 1.96  ± 0.84 | 51 | 1.73  ± 0.72 | -1.38 | 0.167 |
| I feel confident using the guideline in print format/MAGICapp.^b^ | 52 | 3.08  ± 1.20 | 51 | 2.41  ± 1.04 | -2.99 | 0.003* |
| I consider the guideline in print format/MAGICapp to be useful. ^b^ | 52 | 3.69  ± 1.06 | 51 | 3.86  ± 0.92 | -1.17 | 0.240 |
| The guideline in print format/MAGICapp facilitates my daily work. ^b^ | 52 | 3.06  ± 1.15 | 51 | 3.10  ± 1.25 | -0.42 | 0.676 |
| **User-friendliness of guideline** |  |  |  |  |  |  |
| Total (sum user-friendliness) | 52 | 22.67  ± 6.94 | 51 | 24.20  ± 5.70 | -1.76 | 0.078 |
| I can well imagine using the guideline in print format/MAGICapp on a regular basis. ^d^ | 52 | 2.27  ± 1.11 | 51 | 2.55  ± 1.01 | -1.76 | 0.078 |
| I find the guideline in print format/MAGICapp unnecessarily complex. ^e^ | 52 | 2.08  ± 1.01 | 51 | 2.31  ± 0.88 | -1.60 | 0.109 |
| I find the guideline in print format/MAGICapp easy to use. ^d^ | 52 | 2.17  ± 0.96 | 51 | 2.51  ± 0.73 | -2.36 | 0.018* |
| I think that I would need support (contact person) to use the guideline in print format/MAGICapp. ^e^ | 52 | 2.67  ± 1.08 | 51 | 2.20  ± 0.98 | -2.60 | 0.009* |
| I think that the various functions of the guideline are well integrated in the print format/MAGICapp. ^d^ | 52 | 1.98  ± 0.83 | 51 | 2.61  ± 0.83 | -3.72 | <0.001* |
| I find that there are too many inconsistencies in the guidelines in print format/MAGICapp. ^e^ | 52 | 2.44  ± 0.73 | 51 | 2.55  ± .67 | -1.03 | 0.304 |
| I can imagine that most people quickly learn to work with the guideline in print forma/MAGICapp. ^d^ | 52 | 2.08  ± 1.01 | 51 | 2.49  ± 0.86 | -2.55 | 0.011* |
| I find the handling in print format/MAGICapp very complicated. ^e^ | 52 | 2.17  ± 1.12 | 51 | 2.49  ± 0.93 | -1.63 | 0.104 |
| I felt very confident using the guideline in print format/MAGICapp. ^d^ | 52 | 2.19  ± 0.99 | 51 | 2.16  ± 0.73 | -0.44 | 0.659 |
| I had to learn a lot of things before I could work with the guideline in print format/MAGICapp. ^e^ | 52 | 2.62  ± 1.12 | 51 | 2.33  ± 0.93 | -1.76 | 0.078 |

IG: Intervention group, T1: Post-intervention survey, N: number (group size), M: mean, SD: Standard deviation, ^a^: 5-point Likert scale: 1=never, 5=daily, ^b^: 5-point Likert scale: 1=strongly disagree, 5=strongly agree, ^d^: 5-point Likert scale: 0=strongly disagree, 4=strongly agree, ^e^: 5-point Likert scale: 0=strongly agree, 4:=strongly disagree, p_b_: p-value from Mann-Whitney U-test; * p < 0.005
